# Muscle dynamics analysis by clustered categories during jogging in patients with anterior cruciate ligament deficiency

**DOI:** 10.1186/s12891-023-07000-w

**Published:** 2023-11-28

**Authors:** Haoran Li, Hongshi Huang, Si Zhang, Shuang Ren, Qiguo Rong

**Affiliations:** 1 Department of Mechanics and Engineering Science, College of Engineering, Peking University, Beijing, 100871 China; 2 Department of Sports Medicine, Peking University Third Hospital, Institute of Sports Medicine of Peking University, Beijing, 100871 China

**Keywords:** Anterior cruciate ligament deficiency, Clustering, Dynamics, Muscle force, Gait analysis

## Abstract

**Background:**

Patients with anterior cruciate ligament (ACL) deficiency (ACLD) tend to have altered lower extremity dynamics. Little is known about the changes in dynamic function and activation during jogging in patients with ACLD.

**Methods:**

Twenty patients with an injured ACL before ACL reconstruction (ACLD group) and nine healthy male volunteers (control group) were recruited. Each volunteer repeated the jogging experiment five times. Based on the experimental data measured, a musculoskeletal multibody dynamics model was employed to simulate the tibiofemoral joint dynamics during jogging. Eighteen muscles were used for analysis. The obtained dynamics data were used for clustering and curve difference analysis.

**Results:**

The 18 muscles studied were divided into 3 categories. All the quadriceps, the soleus, the gastrocnemius, and the popliteus were classified as label 1. All the hamstrings were classified as label 2, and the sartorius muscles were classified as label 3. Among them, the classification of the short head of the biceps femoris was significantly different between the two groups (*P* < 0.001). The force curves of all 18 muscles and the between-group differences were studied according to clustered categories. Most muscle force in label 1 was approaching zero in the terminal stance phase, which was significantly lower than that in the control group (*P* < 0.05). The muscle force in label 2 had areas with significant differences in the stance phase. Muscle force in label 3 was significantly lower than that in the control group in the pre-swing phase.

**Conclusions:**

This study showed that there are various changes of muscle function and activation in patients with ACLD. Through clustering and curve analysis, the joint reactions and changes of different muscle forces in the gait cycle between the ACLD and control groups could be further clarified.

## Background

The knee is the largest and most complex joint in the human body. The anterior cruciate ligament (ACL) is important in the structure of the knee joint. The ACL limits the anterior displacement and internal rotation of the tibia and restricts the varus and valgus of the joint. Thus, it is a substantial contributor to the stability of the knee.

ACL rupture is one of the most common sports-related injuries. A 14-year follow-up study [1] and a biomechanical measurement study [2] also demonstrated that patients have a high risk of osteoarthritis even after treatment and ACL reconstruction. After suffering from ACL deficiency (ACLD), the kinematics and dynamics of the knee always change badly. Zhang et al. [3] studied the gait of patients with ACLD with different meniscus tear states. They found that different patients had different walking gait patterns and different knee flexion angles. Many studies have investigated the kinematics and dynamics in ACLD-affected knees. Some showed that when walking on a horizontal surface, patients adopted quadriceps avoidance [4] and stiffening strategy [5, 6] gait patterns. The patterns indicated reductions in the knee flexion moment and peak knee flexion angle. Ren et al. [6] further studied the kinematics and dynamics of patients with ACLD with meniscus injuries when waking horizontally. However, due to the difficulty in directly measuring the force, most studies of the knee have been limited to kinematics analysis, for example by measuring kinematics to investigate basic gait patterns in adolescents with autism spectrum disorder [7], children with Achondroplasia [8], and the older adults [9]. Only a few studies [10–12] have focused on the changes of dynamics in patients with ACLD during jogging.

It is difficult to measure the knee joint dynamics, regardless of whether in vivo or in vitro [13, 14]. Musculoskeletal modeling has emerged in recent years and can resolve these limitations. Such modeling can simulate the kinematics of the knee joint and even estimate the force and moment of the knee joint with the help of inverse dynamics analysis. It can even estimate muscle forces with optimization methods. Andriacchi and Dyrby [15] used inverse dynamics to analyze the changes in the knee joint force and moment in patients with unilateral ACLD while walking. They determined the phases that led to abnormal ACLD kinematics as the swing phase in the gait cycle.

Few studies have specifically focused on the changes in knee muscles. Hug et al. [16] used machine learning to analyze the electromyogram of motor unit activations. They proved the existence of muscle activation patterns and the uniqueness among different people. Tsepis et al. [17] analyzed the smoothness of isokinetic torque production in ACL-deficient knees. For each extension and flexion frequency domain pattern, the maximum frequency values contained within most of the total signal power levels was calculated. However, few studies have focused on the function and activation analysis of muscle force.

This study was performed to analyze the function and activation of the knee muscle force in patients with ACLD during jogging through clustering and muscle force curve analysis grouped according to the clustering results.

## Methods

### Subjects

Twenty young men with unilateral chronic ACLD (contralateral side intact) were recruited before undergoing ACL reconstruction (ACLD group). The patient’s ACL was completely torn. All patients’ knees had been injured during sports-related activities 6 months to 4 years prior to testing. Exclusion criteria were the existence of no prior ACL and concomitant meniscal and ligament rupture in patients, as well as no history of musculoskeletal disorders of the hip or ankle. Their physical activity level was assessed by the Tegner score, which is a reliable and valid tool for assessing the activity level of patients with ACLD [18]. The mean activity level of all patients was normal before the knee injury (score range 3.0–6.0). Nine healthy men volunteered for this study (control group). Ethics approval was obtained from the university’s ethics committee, and written informed consent was obtained from all subjects. All participants were male because biomechanical characteristics differ between sexes [11]. The morphological data are shown in Table 1. No significant differences were found in age, height, weight, body mass index, or pace between the two groups. For most participants, the time since injury exceeded 6 months.

**Table 1:** Characteristics of participants in each group

Parameters	Control	ACLD
Age (years)	29.22 ± 5.61	27.6 ± 4.07
Height (cm)	173.67 ± 1.95	176.3 ± 6.06
Weight (kg)	74.06 ± 4.73	77.85 ± 8.17
BMI (kg/m2)	24.56 ± 1.62	25.08 ± 2.64
Pace (m/s)	2.32 ± 0.17	2.35 ± 0.27
Time since injury (months)	/	10.73 ± 7.09
Tegner score	/	4.15 ± 1.73

Data are presented as mean ± standard deviation

*ACLD* anterior cruciate ligament deficiency, *BMI* body mass index

### Data collection and modeling analysis

**Fig. 1:**
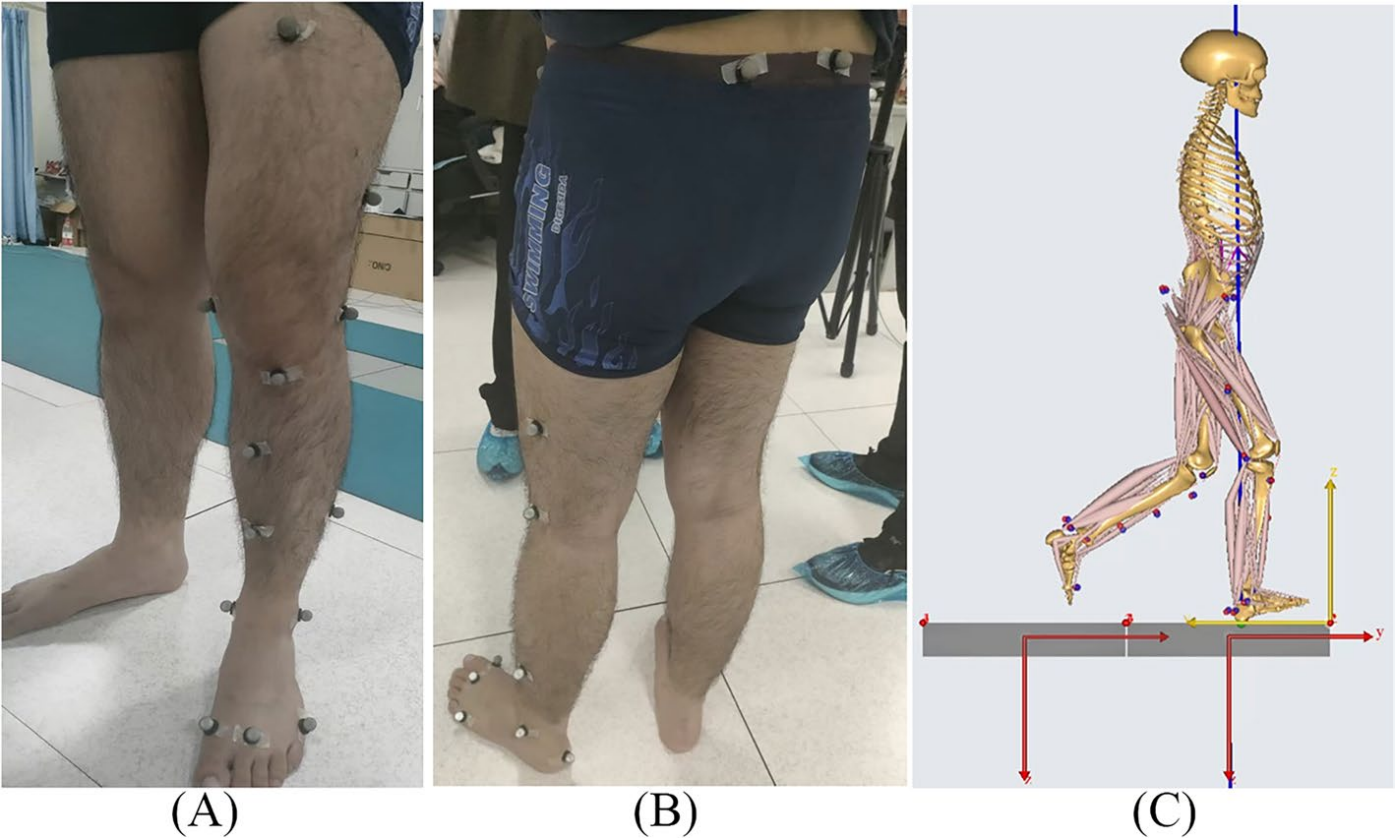
**A** The front view and **B** side view of the placement of the experimental markers; **C** The side view of locations of the designated (red)/experimental (blue) markers on the musculoskeletal model when performing inverse dynamic analysis and muscle force prediction

From January 2014 to December 2016, the experimental data were collected during jogging using an optical motion capture system (Vicon MX; Oxford Metrics, Yarnton, Oxfordshire, UK). Ground reaction forces were measured using two 1000 Hz embedded force plates (Advanced Mechanical Technology Inc., Watertown, MA, USA). The marker trajectory data were filtered at 12 Hz, and the force data were filtered using a low-pass Butterworth filter at 100 Hz. A series of 14 mm markers were attached to the anatomical lower limbs at specific locations based on the plug-in-gait model to track the segmental motion during jogging (Fig. 1). The participants were asked to run along a 10-m path at a self-selected speed, and the kinematic data were recorded by eight cameras. No participants reported pain during jogging. For each subject, five successful jogging trials were recorded, and these results were imported into the multi-body dynamics software AnyBody Modeling System version 6.0.5 (AnyBody Technology, Aalborg, Denmark) to estimate the kinetics of the knee joint.

A generic lower extremity model [19] implemented in the AnyBody Modeling System was employed for the analysis. This model comprised 12 body segments, and 11 joints were used to connect the segments. Six joint degrees of freedom were considered for each leg, with a spherical joint with three degrees of freedom for the hip joint and a universal joint with two degrees of freedom for the ankle joint. The knee joint was modeled as a hinge joint with one degree of freedom because of the soft tissue artifact error [20]. Based on the morphological parameters measured from each subject, each model was scaled with a mass-fat scaling algorithm to perform the subject-specific jogging simulation. The inverse kinematics based on the least squares was used to compute the kinematics of the simulation. The inverse dynamics analysis was used to determine the kinetics of the lower limb extremity based to kinematics and ground reaction forces. The dynamics of the model were provided by Hill-type muscle actuators. The min/max recruitment principle solver [21], which has good numerical convergence and physiological representation, was used to predict the muscle force after the inverse dynamics analysis.

$$minimize\ max \left(\frac{{f}_{i}^{(M)}}{{N}_{i}}\right)$$

$$\text{subject to}\quad \mathbf{C}\mathbf{f}=\mathbf{d},\quad 0\le {f}_{i}^{\left(M\right)}\le {N}_{i},\quad i\epsilon \left\{1, \cdots ,{n}^{(M)}\right\}$$

where ${n}^{(M)}$ is the number of muscles, ${f}_{i}^{\left(M\right)}$ is the respective muscle force, ${N}_{i}$ refers to the strength of the muscle. $\mathbf{f}$ contains all unknown muscle forces. $\mathbf{d}$ contains all known forces. $\mathbf{C}$ is the coefficient-matrix of moment arms.

### Muscle data processing

After the inverse dynamics data were calculated using AnyBody, the data of 18 muscles needed to be output. According to the characteristics of the model and anatomy related to the knee muscles, the output muscle force data were as follows: rectus femoris (RF), popliteus (POP), vastus lateralis superior (VLS), vastus medialis inferior (VMI), vastus medialis mid (VMM), vastus lateralis inferior (VLI), vastus medialis superior (VMS), vastus intermedius (VI), gastrocnemius lateralis (GL), gastrocnemius medialis (GM), soleus medialis (SOLm), soleus lateralis (SOLl), semitendinosus (ST), semimembranosus (SM), proximal sartorius (SAp), distal sartorius (SAd), biceps femoris long head (BFlh), and biceps femoris short head (BFsh).

Five different jogging trials were simulated based on the experimental data, and their muscle force average values were used to perform analysis using MATLAB version 2019b (MathWorks, Natick, MA, USA).

The acquired muscle force data needed to be processed in a dimensionless manner. The nondimensionalization of the force data needed to be divided by the subject’s weight (Newtons) [15]. To investigate one gait cycle, the muscle force data were interpolated to 0%–100% gait cycle. Moreover, to more intuitively study the characteristics and patterns of muscle strength, all muscle data were normalized to their maximal muscle force within that cycle, leading to a normalized muscle amplitude between 0 and 1 [16]. The t-test was used to test the differences in muscle force between the ACLD and control groups.

### Data analysis, clustering, and statistics

In this study, the k-means algorithm was adopted for clustering. Cluster analysis is a multivariate technique that was developed to identify categories that may exist naturally [22]. Because no label for classification exists, cluster analysis is an application of unsupervised learning. The main idea of cluster analysis is that the features of objects belonging to the same group are similar, and those belonging to other groups are different. The k-means clustering algorithm is one approach to clustering. It is an iterative optimization algorithm that assigns each object to one of k clusters. Before cluster analysis starts, the number k to be classified and the initial centers of these k groups should be chosen. During the clustering process, the distances between all the points and centers are computed. Each object should be assigned to the closest center according to the distances. The centers of these k groups are then iteratively calculated and the assignments of the objects are iteratively updated until the group assignments no longer change. In general, the principle of the k-means clustering algorithm is to optimize the following objective function to minimize it:

$${\mathbb{P}}=\sum\limits_{n=1}^{N}\sum\limits_{k=1}^{K}{r}_{nk}{D}_{nk},\qquad \mathrm{where}\quad {r}_{nk}=\left\{\begin{array}{lr}1 & when\ n\in k\ group\\ 0 & otherwise\end{array} \right.$$

where n is the nth object, k is the kth group, and ${D}_{nk}$ is the distance from each point to every centroid. In this study, ${D}_{nk}$ was represented by the squared Euclidean distance, calculated as follows:

$${D}_{nk}={\Vert {x}_{n}-{c}_{k}\Vert }^{2}$$

where ${x}_{n}$ represents the data of the nth object ${c}_{k}$ represents the cluster centroid of the kth group. For different datasets, different distance metric methods can also be used. The k-means clustering algorithm in this study was performed in MATLAB.

Normalized data processed as described above were used to assess class and functional differences between different muscles. Two new datasets consisted of an m × n matrix, where m = the number of people in the ACLD group or healthy group × 18 muscles and n = 101 time points. Each row corresponded to the average force of one subject’s one muscle over five trials for one gait cycle. Thus, the ACLD group formed a 360 × 101 matrix and the control group formed a 324 × 101 matrix, in which both of the two healthy legs were used. The 101 time points were used as variables for analysis and to classify all muscles of all participants [16, 23]. The two matrices were clustered separately using the k-means method. The statistical mode of all categories in the ACLD or control group matrix after clustering for each muscle was taken as the final label for that muscle. The sign test was used to compare all the labels of each muscle between the ACLD and control groups.

Each muscle data curve pattern was additionally transformed into the frequency domain via fast Fourier transform using MATLAB (sampling rate = 256). The corresponding frequency of the occurrence of the maximum Fourier coefficient magnitude of each muscle’s frequency domain was recorded. The t-test was used to compare the frequency of the same muscle force between the ACLD and control groups.

## Results

**Fig. 2:**
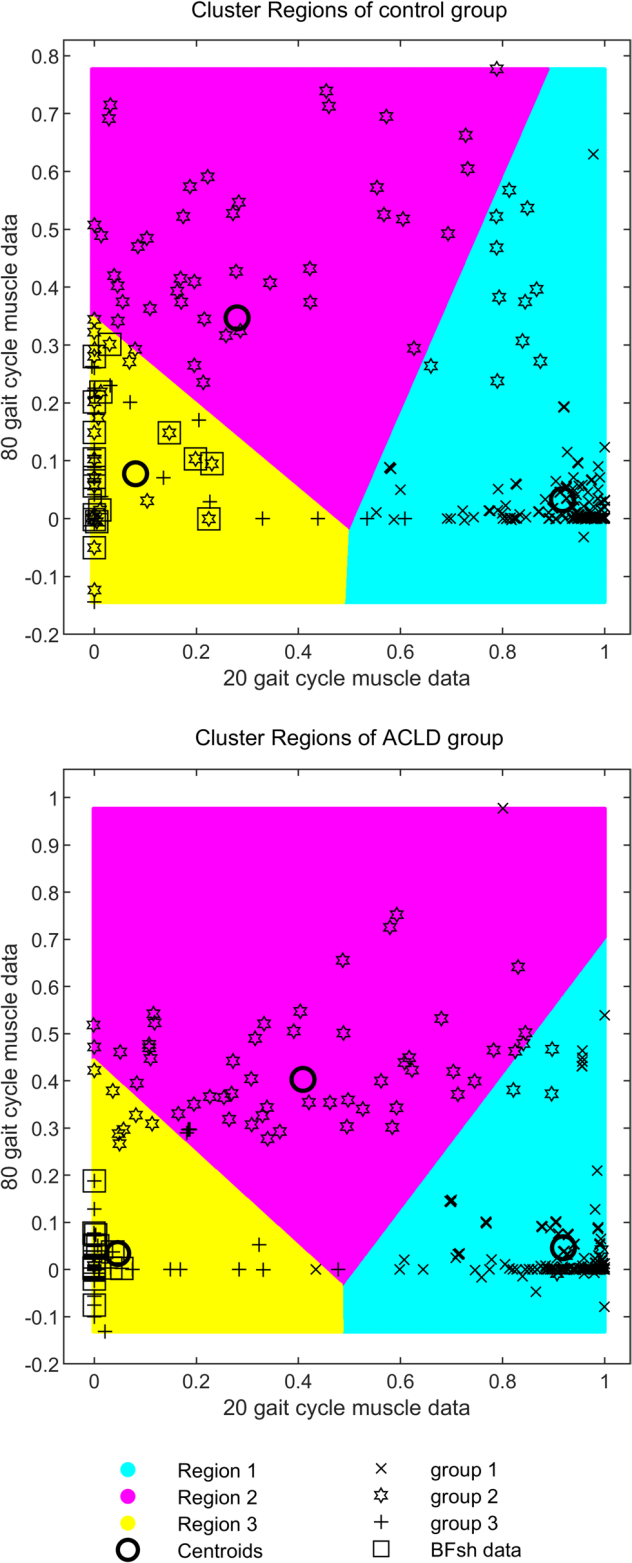
Cluster regions of all data between control group (up) and ACLD group (down). The horizontal axis represents the 20th gait cycle, and the vertical axis represents the 80th gait cycle. The three colors represent the three cluster regions. The points circled by the squares represent the BFsh muscle data with significantly different classifications during the clustering process. ACLD, anterior cruciate ligament deficiency; BFsh, biceps femoris short head

The clustering results in the control group showed that the GL, GM, VLS, VMI, VMM, RF, POP, SOLm, SOLl, VLI, VMS, and VI muscles were classified as label 1 (all the quadriceps, the soleus, the gastrocnemius, and the popliteus). The ST, SM, BFlh, and BFsh muscles were classified as label 2 (all the hamstrings). The SAp and SAd were classified as label 3 (the sartorius muscles). In the ACLD group, the labels of the SAp, SAd, and BFsh muscles partially changed. Among these, the BFsh muscle underwent significant changes (*P* < 0.001), and its category in the ACLD group became label 3. To more intuitively reflect the classification differences of clusters, the cluster regions of the 20th and 80th gait cycles were drawn and the position of the BFsh muscle data was highlighted (Fig. 2). A scatterplot was drawn using the muscle data from the 20th gait cycle as the horizontal coordinate and the data from the 80th gait cycle as the vertical coordinate. Points of different labels were represented by different symbols. The regions of different labels were indicated by color blocks. The highlighted BFsh data were represented by different symbols with different positions between the two groups.

**Fig. 3:**
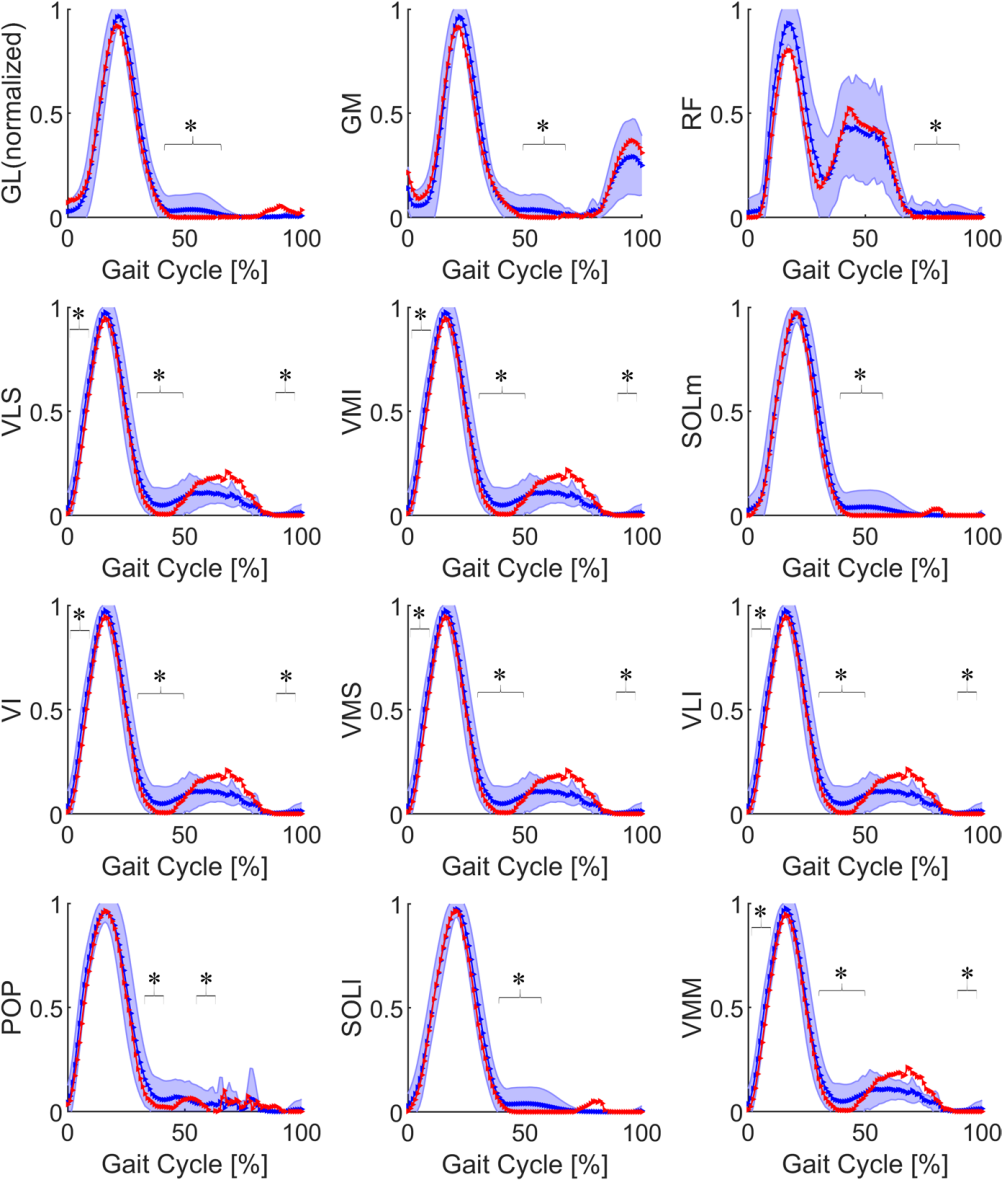
Average force and error band of muscles in label 1 between control group (blue) and ACLD group (red). The blue shaded area represents the mean ± standard deviation of the control group. *Statistically significant difference (*P* < 0.05) between ACLD and control groups. ACLD, anterior cruciate ligament deficiency; GL, gastrocnemius lateralis; GM, gastrocnemius medialis; RF, rectus femoris; VLS, vastus lateralis superior; VMI, vastus medialis inferior; SOLm, soleus medialis; VI, vastus intermedius; VMS, vastus medialis superior; VLI, vastus lateralis inferior; POP, popliteus; SOLl, soleus lateralis; VMM, vastus medialis mid

The average muscle force and the error band of muscles in label 1 are shown in Fig. 3. These muscles were mainly activated in the 0%–40% interval of the gait cycle. The muscle force of most of the muscles in label 1 approached zero in the terminal stance phase, which was significantly lower than in the control group (*P* < 0.05). Because the normalized data of each participant was averaged, there were cases where the peak value was less than 1 in the result, which meant that the peak positions of the muscle forces are different among the participants.

**Fig. 4:**
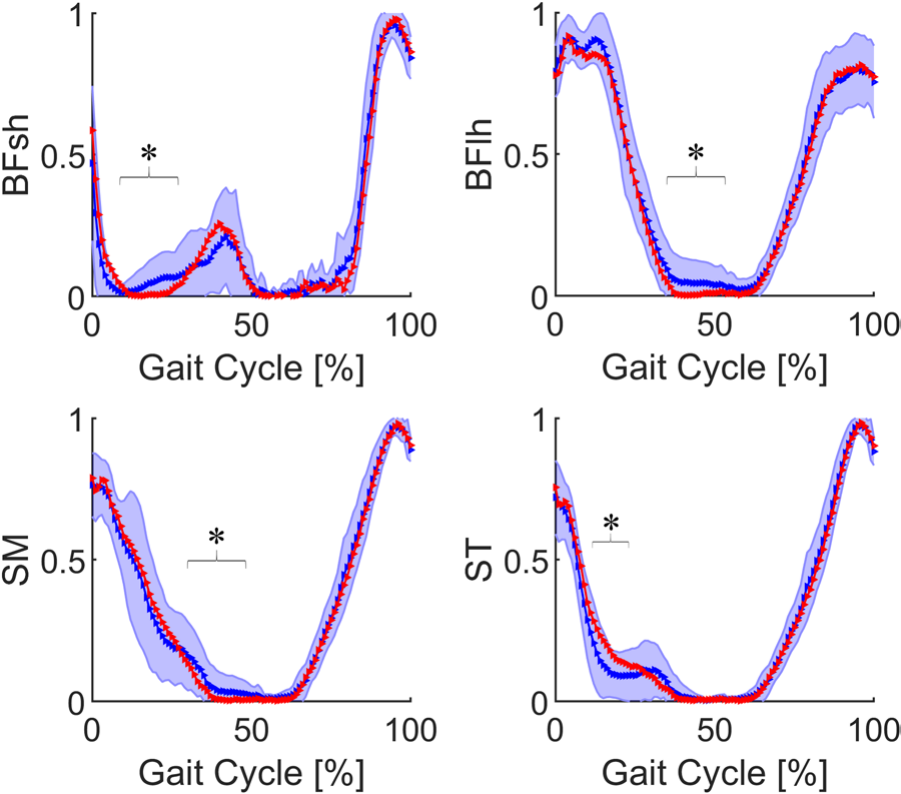
Average force and error band of muscles in label 2 between control group (blue) and ACLD group (red). The blue shaded area represents the mean ± standard deviation of the control group. *Statistically significant difference (*P* < 0.05) between ACLD and control groups. ACLD, anterior cruciate ligament deficiency; BFsh, biceps femoris short head; BFlh, biceps femoris long head; SM, semimembranosus; ST, semitendinosus

The average muscle force and the error band of muscles in label 2 are shown in Fig. 4. These muscles were mainly activated in the swing phase (62%–100% of the gait cycle). The muscle force of BFsh, BFlh, and SM was significantly lower than that in the control group (*P* < 0.05) in different positions of the stance phase, and the muscle force of ST was significantly higher than that in the control group (*P* < 0.05) in 15%–20% of the gait cycle.

**Fig. 5:**
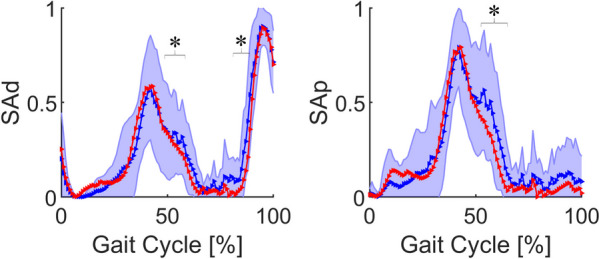
Average force and error band of muscles in label 3 between control group (blue) and ACLD group (red). The blue shaded area represents the mean ± standard deviation of the control group. *Statistically significant difference (*P* < 0.05) between ACLD and control groups. ACLD, anterior cruciate ligament deficiency; SAd, distal sartorius; SAp, proximal sartorius

The average muscle force and the error band of muscles in label 3 are shown in Fig. 5. These muscles were mainly activated in the terminal stance phase (31%–50% of the gait cycle) and the pre-swing phase (50%–62% of the gait cycle). The muscle force of label 3 was significantly lower than that in the control group (*P* < 0.05) in the pre-swing phase.

**Fig. 6:**
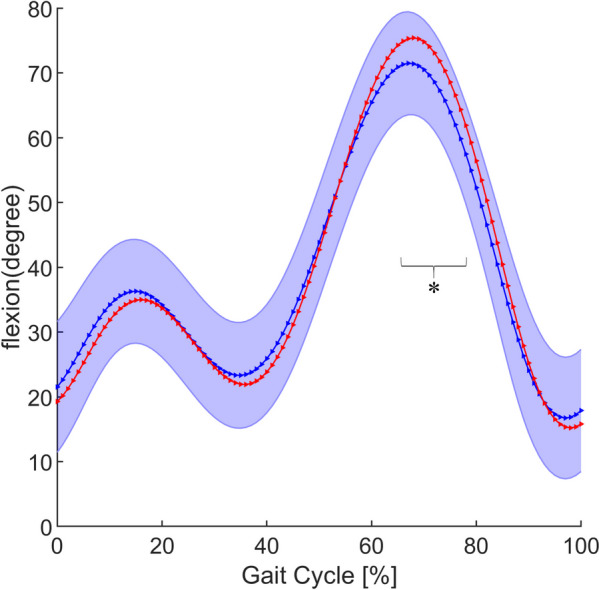
Sagittal tibiofemoral joint kinematics between control group (blue) and ACLD group (red). The blue shaded area represents the mean ± standard deviation of the control group. *Statistically significant difference (*P* < 0.05) between ACLD and control groups. ACLD, anterior cruciate ligament deficiency

To measure whether changes in the calculated muscle forces are meaningful, sagittal tibiofemoral joint kinematics are shown in Fig. 6. Unlike walking, the knee flexion of jogging was significantly different in the initial swing phase (62%–75% of the gait cycle).

**Fig. 7:**
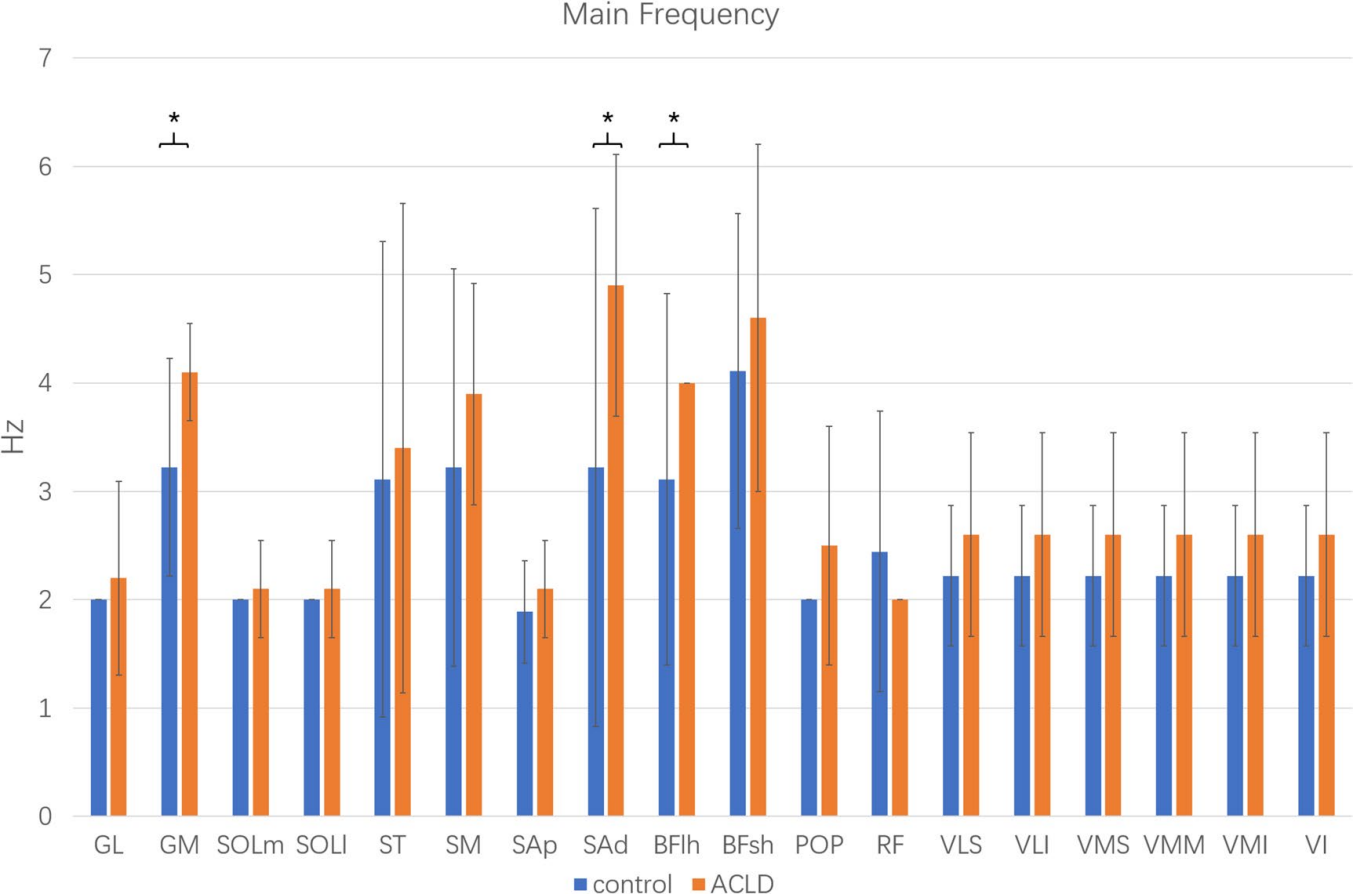
Main frequency of each muscle between ACLD and control groups. *Statistically significant difference (*P* < 0.05) between ACLD and control groups. ACLD, anterior cruciate ligament deficiency; GL, gastrocnemius lateralis; GM, gastrocnemius medialis; ST, semitendinosus; SM, semimembranosus; SAp, proximal sartorius; SAd, distal sartorius; BFlh, biceps femoris long head; BFsh, biceps femoris short head; POP, popliteus; SOLm, soleus medialis; SOLl, soleus lateralis; VLS, vastus lateralis superior; VMI, vastus medialis inferior; VMM, vastus medialis mid; RF, rectus femoris; VLI, vastus lateralis inferior; VMS, vastus medialis superior; VI, vastus intermedius

Of all 18 muscles studied, there were significant differences in the main frequency of GM, SAd, and BFlh between the ACLD and control groups (*P* < 0.05). The dominant frequency and standard deviation of each muscle between the ACLD and control groups are shown in Fig. 7. The main peak position frequencies of the GM, SAd, and BFlh obtained by Fourier spectrum analysis in the ACLD and control groups were 4.1 ± 0.4, 4.9 ± 1.2, and 4.0 ± 0.0 Hz compared with 3.2 ± 1.0, 3.2 ± 2.4, and 3.1 ± 1.7 Hz, respectively.

## Discussion

This study investigated the muscle forces, muscle frequency alterations, and muscle classification in ACLD-affected knees during jogging. According to the principles of anatomy, all 18 muscles involved in the analysis were related to the movement of the knee joint. For the convenience of the following discussion, a prior understanding of how muscles function in relation to the knee is required [24, 25]. The quadriceps femoris plays a role in knee extension in the sagittal plane. The effect of the hamstrings, including the ST, SM and the biceps femoris, are knee flexion in the sagittal plane. The function of the gastrocnemius, soleus, sartorius and POP is knee flexion.

### Classification of muscles

According to the clustering results, the 18 muscles were divided into 3 categories by function and curvilinear shape (Table 2). In the label 1 group, all muscles had peaks at the beginning of the stance phase (Fig. 3), and these muscles were mainly active during this period, different from knee flexion (Fig. 6). All the vastus muscles had the same dynamics and were classified as label 1. They play the most important role in knee strength during the stance phase [26]. The soleus, gastrocnemius, and POP are muscles on the tibia that act against the quadriceps. They are innervated by the tibial nerves. Their most active period is also in the interval of 0%–40% of the gait cycle. Although there were differences in force among the different vastus muscles, and the patients with ACLD exhibited a quadriceps avoidance gait [4, 27], their normalization images were similar (Fig. 3). Knee joint extension is still mainly provided by the quadriceps muscles. Thus, in the ACLD group, the classifications of the muscles in label 1 were not changed.

**Table 2:** Labels of all the muscles in the two groups by clustering

Muscle	Control group	ACLD group	*p*-value
GL	1	1	1
GM	1	1	0.25
ST	2	2	1
SM	2	2	1
SAp	3	3	0.25
SAd	3	3	1
BFlh	2	2	1
VLS	1	1	1
VMI	1	1	1
VMM	1	1	1
RF	1	1	0.125
POP	1	1	1
SOLm	1	1	1
SOLl	1	1	1
BFsh	2	3^*^	< 0.001^*^
VLI	1	1	1
VMS	1	1	1
VI	1	1	1

*ACLD* anterior cruciate ligament deficiency, *GL* gastrocnemius lateralis, *GM* gastrocnemius medialis, *ST* semitendinosus, *SM* semimembranosus, *SAp* proximal Sartorius, *SAd* distal Sartorius, *BFlh* biceps femoris long head, *BFsh* biceps femoris short head, *POP* popliteus, *SOLm* soleus medialis, *SOLl* soleus lateralis, *VLS* vastus lateralis superior, *VMI* vastus medialis inferior, *VMM* vastus medialis mid, *RF* rectus femoris, *VLI* vastus lateralis inferior, *VMS* vastus medialis superior, *VI* vastus intermedius

^*^Statistically significant difference (*P* < 0.001) between ACLD and control groups

All muscles in the label 2 group were hamstrings (located on the femur), which mainly play the role of knee flexion and antagonization of the quadriceps. These muscles’ peaks were primarily at the beginning of the stance phase and the end of the swing phase. In the patients with ACLD, the BFsh was classified as label 3 instead of label 2. This may have been due to the compensatory mechanism and neural control of the muscles in these patients [28, 29]. The ST and biceps femoris were most active in the terminal swing phase. When the ST was not able to generate enough force because of injury-related pain, the biceps femoris compensated [28]. The biceps femoris muscle consists of a long head and a short head that are innervated by the tibial division and common fibular division of the sciatic nerve, respectively [30]. The BFlh passes through the knee joint and the hip joint, but the BFsh only passes through the knee joint. Different origins can produce different force vectors during contraction [31]. The BFsh has clinical significance because it is the only muscle innervated by the fibular portion of the sciatic nerve [30]. With the exception of the BFsh, the muscles on the back of the thigh are mainly hip extensors and knee flexors with subtle rotational features [29]. Therefore, because of the different positions, functions, and innervating nerves of these muscles, as well as the different neural control of the muscles in patients with long-term ACLD, the BFsh also differed in its compensation mechanism in ACLD-affected knees and differed in classifications.

All muscles in the label 3 group were sartorius muscles. They are located on the femur, are innervated by the femoral nerve, and play the role of knee flexion. Their peak position occurred at 40% of the gait cycle (Fig. 5), at which the BFsh also had a small peak. In patients with ACLD, the muscle classifications in the label 3 group did not change, and the BFsh was classified into label 3.

Compared with the other 16 muscles, the frequency of the main peak of the spectrum of GM, SAd, and BFlh showed significant differences between the ACLD and control groups during jogging (Fig. 7). The frequency of extensor muscles such as the vastus did not change significantly, which can be explained by the fact that the muscle activation patterns did not change significantly. Partial changes in muscle force during the gait cycle did not affect these muscle activation patterns. Correspondingly, the activation patterns of antagonistic muscles such as the hamstrings changed significantly in ACLD-affected knees [28]. Therefore, the Fourier frequency analysis results contained only the flexors. In the human body, there is a functional tendoligamentous unit called the arcuate ligament complex, which includes the biceps femoris and gastrocnemius [32]. This structure plays an important role in posterolateral stabilization. In ACLD-affected knees, the arcuate ligament complex changes its activation patterns. Thus, there were significant differences in the frequency of the GM, SAd, and BFlh, between the ACLD and control groups.

### Muscle force curves between ACLD and control groups

The differences in different muscle force curves are shown in Figs. 3, 4 and 5. Figure 3 shows that the muscle force in the ACLD group was significantly reduced, even approaching zero, at 40% of the gait cycle and the other positions, at which the muscle force in the control group was small. This was due to the avoidance strategy of the quadriceps [4, 27]. In ACLD-affected knees, the extensor moment of the knee joint was reduced or even completely eliminated. This is because ACLD leads to poor stability of the knee joint, which requires active reduction of tibial anterior displacement, resulting in adaptive changes in muscle strength [26, 27, 33]. Thus, the soleus, gastrocnemius, and POP decreased and even approached zero at the same position as quadriceps strength reduction.

Among the hamstrings, the ST has the highest muscle activation and is more strongly recruited during jogging than the SM and biceps femoris [28]. From an anatomical perspective, Lempainen et al. [29] argued that each hamstring has a unique anatomy that should be considered individually when studying injuries. The biceps femoris is activated in the mid-swing phase, and the ST is primarily active in the terminal swing phase. Therefore, Fig. 4 shows that the peak of the ST occurred in the interval of 87%–100% of the gait cycle and that the peak of the biceps femoris occurred in the interval of 75%–87%. In ACLD-affected knees, the quadriceps avoidance pattern does not fully solve the problems of tibial anterior translation [27]. Many studies have shown that hamstring activation can reduce tibial anterior displacement in ACLD-affected knees and maintain knee stability [27, 33–35]. Thus, compared with the control group, the muscle force of the ST in ACLD-affected knees significantly increased in the mid-stance phase. Additionally, the normalized muscle force of the BFlh and SM in the terminal stance phase significantly decreased to zero. This can be explained by the quadriceps also decreasing to zero in the same time period.

Compared with the control group, the sartorius muscle force in the ACLD group decreased significantly during the pre-swing phase (Fig. 5). The small wave peak of the curve in the pre-swing phase also disappeared. This was probably due to the synergy and compensation of other muscles [28]. Meanwhile, in the ACLD-affected knees, the muscle force curve of the BFsh (Fig. 4) was close to that of the sartorius muscle, especially when the muscle force of the BFsh decreased to zero in the mid-stance phase.

Some limitations of this study should be noted. First, some patients with ACLD also had meniscus injuries. Some studies (taking a study from the United States as an example [36]) have shown that about 40% to 80% of patients with ACLD have a concurrent meniscal injury. Compared with patients who have ACLD alone, those with ACLD combined with a meniscus injury have different kinematics and dynamics [6]. Second, the principles of muscles were researched only by knee dynamics. It is necessary to add analysis of the hip joint to further confirm what causes the changes in muscle behavior. Third, electromyography (EMG) data can be introduced. EMG can assist in validating and verifying the muscle forces obtained from the calculations [37, 38].

## Conclusion

Clustering and curve analysis revealed differences in the function and activation of knee muscle force between patients with ACLD and healthy people. The most significant difference in classification of muscles was the BFsh. Through the curve analysis of different groups, we further clarified the changes in different muscle forces in the gait cycle of patients with ACLD. This can be helpful for further study of muscle mechanisms and detection of ACLD.

## Data Availability

The datasets used and/or analysed during the current study are available from the corresponding author on reasonable request.
